# CRKL is a critical target of Hh‐GLI2 pathway in lung adenocarcinoma

**DOI:** 10.1111/jcmm.16592

**Published:** 2021-06-02

**Authors:** Xiaoming Liu, Yan Hu, Bentong Yu, Kai Peng, Xin Gan

**Affiliations:** ^1^ Department of Thoracic Surgery The First Affiliated Hospital of Nanchang University Nanchang China; ^2^ Department of Orthopedics The First Affiliated Hospital of Nanchang University Nanchang China; ^3^ Department of Respiratory and Critical Care The First Affiliated Hospital of Nanchang University Nanchang China

**Keywords:** CRKL, GLI2, Hh, LUAD

## Abstract

Lung adenocarcinoma (LUAD) is one of the important components of non‐small‐cell lung cancer (NSCLC) and leads to many deaths every year. During the initiation and progression of the LUAD, the Hh‐GLI2 pathway plays critical roles. Several components of this pathway have been shown to be amplified or overexpressed in LUAD, providing this pathway as an attractive target for therapeutics. However, a gap in our understanding of the Hh‐GLI2 pathway is the identity of transcriptional targets of GLI2 that drive LUAD tumorigenesis. Here, we show that the oncogene CRKL is a direct target of GLI2. GLI2 turns on *CRKL* transcription through binding its second intron. Furthermore, CRKL is an essential mediator for GLI2‐driven proliferation and migration of LUAD cells. Depletion of CRKL blunts Hh‐GLI2 pathway‐mediated cell proliferation and invasion. Lastly, we find that CRKL knockout cells are more sensitive to EGFR‐TKI and chemotherapeutics. Taken together, our work here identifies a specific target for Hh‐related malignancies and provides CRKL as a promising therapeutic target for LUAD.

## 1 INTRODUCTION

Lung cancer is now ranked the first‐most wide‐ranging cancer and is the leading cause of cancer‐related deaths globally, accounting for near 30% of all cancer‐related mortalities.^1^ Most lung cancers (more than 80%) are non‐small‐cell lung cancers (NSCLCs), which are classified into three subfamilies: adenocarcinoma, squamous cell carcinoma and large cell carcinoma.^2^ In past decades, despite significant advancements in diagnosis and therapy of NSCLC, the long‐term survival rate of patients still remains at a low level.^3^ In recent years, targeted therapy has brought hope to NSCLC patients. Thus, it is urgent to identify the therapeutic target for NSCLC.

During NSCLC initiation and progression, Hh pathway plays an important role.^4^ Smoking, the main cause of NSCLC, also activates Hh pathway to promote NSCLC cell proliferation and migration.^5^ The overactivation of Hh pathway is closely linked with NSCLC occurrence.^6^ Besides, Hh pathway is also indispensable in embryonic development, organogenesis and adult stem cell maintenance.^7^, ^8^, ^9^ Blockade of Hh pathway possibly leads to developmental defect and adult stem cell lost. Therefore, treatment the NSCLC patients with Hh pathway inhibitors will bring serious adverse effects. Although several targets of Hh‐GLI2 pathway have been reported,^10^, ^11^ identification of *bona fide* effectors downstream of GLI2 would not only deepen insights of the oncogenic mechanisms of this pathway, but also provide novel actionable target for Hh‐driven cancer.

The adaptor CRKL (v‐CRK avian sarcoma virus CT10 oncogene homolog‐like) is a CRKL like oncogene, which encodes a SH2 and SH3 domain‐containing adaptor.^12^ CRKL could form multiple complexes by interaction with distinct proteins to respond to both extracellular and intracellular stresses.^13^ Many oncogenic stimuli induce CRKL complex formation to activate tumorigenesis‐related signalling pathways.^14^ Thus, CRKL plays oncogenic roles in various types of cancer. As a matter of fact, several studies have clearly demonstrated that CRKL is amplified in NSCLC and acts as an oncogene.^15^, ^16^ However, how CRKL expression is regulated in NSCLC is still elusive.

In this study, we firstly found that GLI2 and CRKL showed accordant up‐regulation in LUAD cells, compared with normal lung epithelial cells. Next, we revealed that GLI2 could activate *CRKL* expression through binding *CRKL* intron region. Then, depletion of CRKL blunted GLI2‐mediated proliferation, migration and invasion in LUAD cells. Furthermore, we uncovered that knockout of CRKL in the LUAD cell enhanced its sensitivity to chemotherapies. In sum, this study has demonstrated that CRKL is a critical target of Hh‐GLI2 pathway in LUAD tumorigenesis and provided CRKL as a promising therapeutic target for LUAD.

## 2 MATERIALS AND METHODS

### 2.1 Cell culture, transfection and Western blot

All cell lines used in this study were purchased from ATCC and cultured as following: A549 cells with F‐12K medium (Gibco) containing 10% FBS (Gibco) and 1% penicillin/streptomycin (Sangon Biotech), H1299, H1975 and H1650 with RPMI‐1640 medium (Gibco) containing 10% FBS and 1% penicillin/streptomycin, 16HBE cells with DMEM (Gibco) medium containing 10% FBS and 1% penicillin/streptomycin. Cell transfection with plasmids or siRNAs was carried out as previously described.^17^ The antibodies used for Western blot were as follows: mouse anti‐CRKL (1:1000; Santa Cruz); mouse anti‐Flag tag (abbreviation for Fg; 1:5000; Sigma); rabbit anti‐GLI2 (1:1000; ABclonal); and mouse anti‐ACTIN (1:5000; Genscript). The siRNAs sequences were shown as follows: *MOCK*‐siRNA, 5′‐CAA ACA CUU CCU UGG AAU GdTdT‐3′; *CRKL*‐siRNA‐1, 5′‐GCU CUG CUC UAC CAU GUU UdTdT‐3′; *CRKL*‐siRNA‐2, 5′‐CGT GAA AGU CAC AAG GAU GdTdT‐3′; *GLI2*‐siRNA‐1, 5′‐GUU CCU CAC GGC GUC GUA GdTdT‐3′; *GLI2*‐siRNA‐2, 5′‐CAA GAC CGA GCC UGA GGG CdTdT‐3′. For chemical treatment, cells were seeded into 96‐well plate at 1 × 10^4^/well. After 24 hours, cells were treated with 5 μg/mL adriamycin (Sigma), 20 μg/mL 5‐FU (Sigma), 10 μg/mL cisplatin (Sigma), 20 μmol/L cyclopamine (MedChemExpress), 10 μmol/L gefitinib (MedChemExpress) or 10 μmol/L GANT61 (MedChemExpress) for additional 24 hours.

### 2.2 In vitro tumorigenicity assay

Cell viability was assessed using MTT assay and clone formation assay. For MTT assay, log‐phase cells were seeded into 96‐well plates. After indicated time, 10 μL MTT (5 mg/mL) was added into each well, followed by incubation for 4 hours before discarding the supernatants. Wash the cells with PBS for three times and add 100 μL DMSO in each well to dissolve crystals for 10 minutes. The absorbance on 490 nm was measured using microplate reader (BioTek). For clone formation assay, 1000 cells were seeded into 6‐well plates for growth additional 2 weeks. The fresh medium was supplemented each 3 days. Then, clones were washed with PBS, fixed with 70% ethanol for 15 minutes and stained with 2% crystal violet (Sangon Biotech). The numbers of clones were counted under a microscope.

Cell migration was tested using wound healing assay. After indicated treatments, equivalent cells were seeded into 6‐well plates with 1% FBS. One yellow pipette tip was used to make a straight scratch. The width of wound was measured at 48 hours and normalized with starting time point.

Cell invasion was examined by transwell assay. After indicated transfections, equivalent cells were seeded on the top of a thick layer of Matrigel (BD Biosciences) in transwell inserts (Corning) and cultured for additional 24 hours. Invasive cells adhered to the lower surface of the filter were washed with PBS, fixed with 4% paraformaldehyde and stained with 2% crystal violet (Sangon Biotech). Numbers of invasive cells were counted under the light microscope.

### 2.3 RNA extraction and real‐time PCR

Total RNA from cultured cells was extracted using TRIzol reagent (Invitrogen). High‐capacity cDNA reverse transcription kit (Applied Biosystems) was used for cDNA synthesis. RT‐PCR was conducted on a CFX96^™^ (Bio‐Rad) with SYBR Green RT‐PCR reagents (Applied Biosystems). The 2‐ΔΔCt method was used for relative quantification. The primer pairs used were as follows: *CRKL*, 5′‐CTG TCG GTG TCC GAG AAC TC‐3′ (forward) and 5′‐ATT GGT GGG CTT GGA TAC CTG‐3′ (reverse); *GLI2*, 5′‐ACA GCA GCC CCA CGC TCT CC‐3′ (forward) and 5′‐AGC GCC CCC GCT CTG CAT C‐3′ (reverse); *PTCH1*, 5′‐GAA GAA GGT GCT AAT GTC CTG AC‐3′ (forward) and 5′‐GTC CCA GAC TGT AAT TTC GCC‐3′ (reverse); *BCL2*, 5′‐CTC AGC AGG TAT CAC ATG GGG‐3′ (forward) and 5′‐CCA AGG TCT TGC GTA CAA ATT CC‐3′ (reverse); *ACTIN*, 5′‐GAT CAT TGC TCC TCC TGA GC‐3′ (forward) and 5′‐ACT CCT GCT TGC TGA TCC AC‐3′ (reverse).

### 2.4 Luciferase assay

200‐bp sequences named with E1, E2, E3, E4 and E5 were respectively subcloned into pGL3‐Basic‐Luc vector (Promega) to generate corresponding luciferase reporter constructs. Meanwhile, the potential GLI2 binding site was mutated (GCG ACC AC to AAA AAA AA) to make E3m‐Luc reporter. The luciferase reporter constructs were co‐transfected with Renilla luciferase plasmid (Promega) into A549 cells. Dual Luciferase Reporter Assay System (Promega) was employed to check the luciferase activity after 24 hours according to the manufacturer's instruction. All luciferase activity data are presented as means ± SD from at least three independent experiments.

### 2.5 Chromatin immunoprecipitation assay

Chromatin immunoprecipitation (ChIP) assay was performed using ChIP‐IT Express Enzymatic Shearing Kit (Active Motif) according to its instructions. A549 cells were cross‐linked with 1% formaldehyde and incubated in enzymatic shearing cocktail dissolved in digestion buffer supplemented with PMSF. The shearing reaction was stopped by adding ice‐cold EDTA and chilling on ice for 10 minutes. Then, the immunoprecipitation was carried out with rabbit anti‐GLI2 antibody or rabbit IgG. The immunoprecipitated DNA was measured using PCR or RT‐PCR. Primers used in ChIP assay were as follows: *CRKL*‐E1, 5′‐ACT GGG CCA CCT CGG CCC ATC‐3′ (forward) and 5′‐TGC CGG GTC TCC CAG CGC TG‐3′ (reverse); *CRKL*‐E2, 5′‐AGT GAT AAA GAA TGG TGG TTAC‐3′ (forward) and 5′‐GCG CAC GCC ACC ATG CCT GG‐3′ (reverse); *CRKL*‐E3, 5′‐TTC ACC ATG TTG GCC AGG CTG‐3′ (forward) and 5′‐GGT AAT TAA AAT GTT CAA GTT C‐3′ (reverse); *CRKL*‐E4, 5′‐ATA AGT CTT TCC ATC CAT TAA C‐3′ (forward) and 5′‐CTT GAA CCA GGG AGG TGG AG‐3′ (reverse); *CRKL*‐E5, 5′‐TAA TGG TCA TTT GGG AAA AC‐3′ (forward) and 5′‐GCC ACT TGT GGG TGC TAC GC‐3′ (reverse).

### 2.6 Cas9‐mediated CRKL knockout

To knock out the endogenous *CRKL*, we used CRISPR/Cas9 tool as previously described.^17^ The sgRNA targeting *CRKL* was GGA CCG CTC CGC CTG GTA TAT GG. It was annealed to the complementary oligo and cloned into pGL3‐U6‐sgRNA‐PGK‐puromycin vector (Addgene). A549 and H1299 cells were co‐transfected with this plasmid and pST‐NLS‐Cas9 plasmid (Addgene). 48 hours after transfection, the cells were treated by puromycin (0.02 mg/mL, Invivogen) and blasticidin (0.75 mg/mL, Invivogen) for additional 48 hours. After cells forming colonies, pick the small colonies into 96‐well plates. Genomic DNA from the cells is amplified by PCR. Putative mutants were further validated by sequencing.

### 2.7 GLI2 and CRKL gene expression analysis

We downloaded gene expression data of previous study from GEO database (GSM1208622).^18^ Next, *GLI2* and *CRKL* expression of all NSCLC samples was extracted to carry out Spearman correlation analysis. Correlation coefficient and *P*‐value were reported.

### 2.8 Statistical analysis

All statistical analysis was performed by GraphPad Prism software. The reported data are representative of at least three independent experiments. For statistical significance, one‐way ANOVA test was applied. *P‐*value less than .05 was considered statistically significant and the *P*‐value less than .001 was considered highly significant. In this study, exact *P*‐values were not shown; statistical significance was as follows: *P* > .05 (ns, no significance), *P* < .05 (*), *P* < .01 (**) and *P* < .001 (***).

## 3 RESULTS

### 3.1 *CRKL* is a target gene of GLI2 in LUAD cells

**FIGURE 1:**
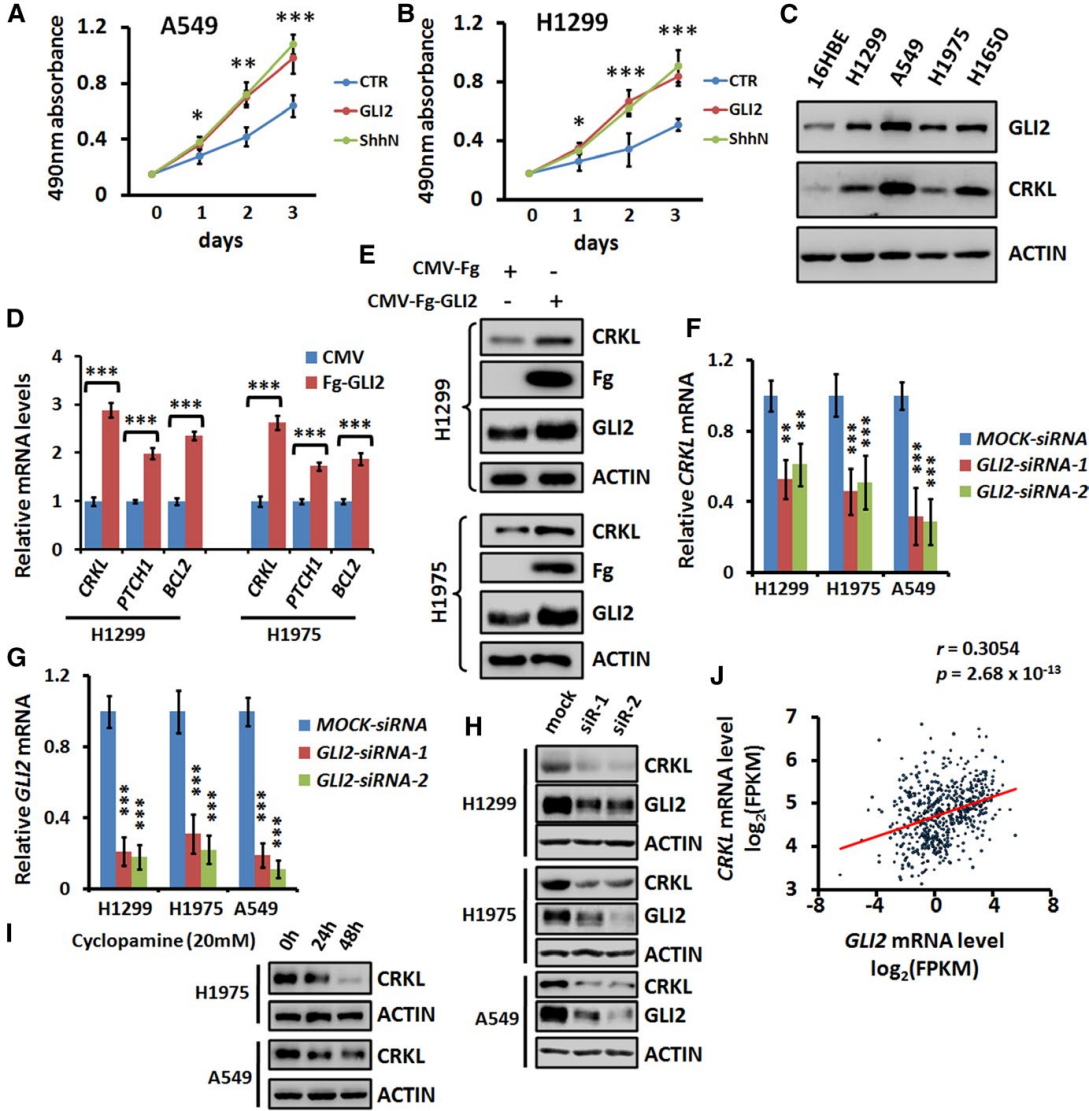
CRKL is a target gene of GLI2. A, 3‐day MTT proliferation results of A549 cells transfected with GLI2 or ShhN. Of note, both GLI2 and ShhN promoted cell proliferation. B, 3‐day MTT proliferation results of H1299 cells transfected with GLI2 or ShhN. C, Western blot results of several LUAD cell lines and normal human bronchial epithelial cell to show the expression of GLI2 and CRKL. ACTIN acts as a loading control. D, Real‐time PCR results of LUAD cells transfected with indicated constructs. Overexpression of GLI2 apparently increased *CRKL* mRNA levels in H1299 and H1975 cells. *PTCH1* and *BCL2* act as positive controls. E, Overexpression of GLI2 increased CRKL protein level in H1299 cells and H1975 cells. F, Real‐time PCR data of LUAD cells treated with indicated siRNAs. Notably, knockdown of GLI2 decreased *CRKL* mRNA levels. G, *GLI2*‐siRNA efficiently silenced endogenous *GLI2* expression. H, Knockdown of GLI2 decreased CRKL protein level in H1299, H1975 and A549 cells. ACTIN acts as a loading control. I, Cyclopamine treatment decreased CRKL protein in H1975 and A549 cells. ACTIN acts as a loading control. J, Spearman correlation analysis of *GLI2* expression and *CRKL* expression in NSCLC patient samples. *GLI2* and *CRKL* showed positive correlation in NSCLC samples. n = 540, *r* = .3054, *P* = 2.68E‐13. All values are mean ± SD (n = 3, *P < .05, **P < .01 and ***P < .001)

It is well known that the hyperactivation of Hh pathway tightly links with LUAD tumorigenesis, but which target gene governs this process is still unclear. At first, we employed two LUAD cell lines to validate that GLI2 or ShhN indeed promoted cell viability (Figure 1A,B), suggesting that these cells could respond to Hh pathway. To identify the critical target gene of GLI2, we analysed the previous ChIP‐seq result and found that *CRKL* is a potential target of GLI2.^18^ In addition, GLI2 positively correlated with CRKL, both showing increased expression in human LUAD cell lines compared with human bronchial epithelial cell (Figure 1C). Consistently, overexpression of GLI2 in NSCLC cells substantially increased *CRKL* mRNA (Figure 1D) and CRKL protein (Figure 1E). In contrast, knockdown of GLI2 apparently decreased *CRKL* mRNA (Figure 1F,G) and CRKL protein (Figure 1H). Furthermore, treatment with cyclopamine, a well‐known Hh pathway inhibitor, also down‐regulated CRKL protein (Figure 1I). Importantly, we found that the level of *CRKL* transcript was positively correlated with *GLI2* mRNA abundance in a large cohort of NSCLC samples (Figure 1J). Taken together, our findings strongly suggest that *CRKL* is a potential target of GLI2 in both LUAD cell lines and NSCLC samples.

### 3.2 GLI2 binds directly to the 2nd intron of *CRKL* gene

**FIGURE 2:**
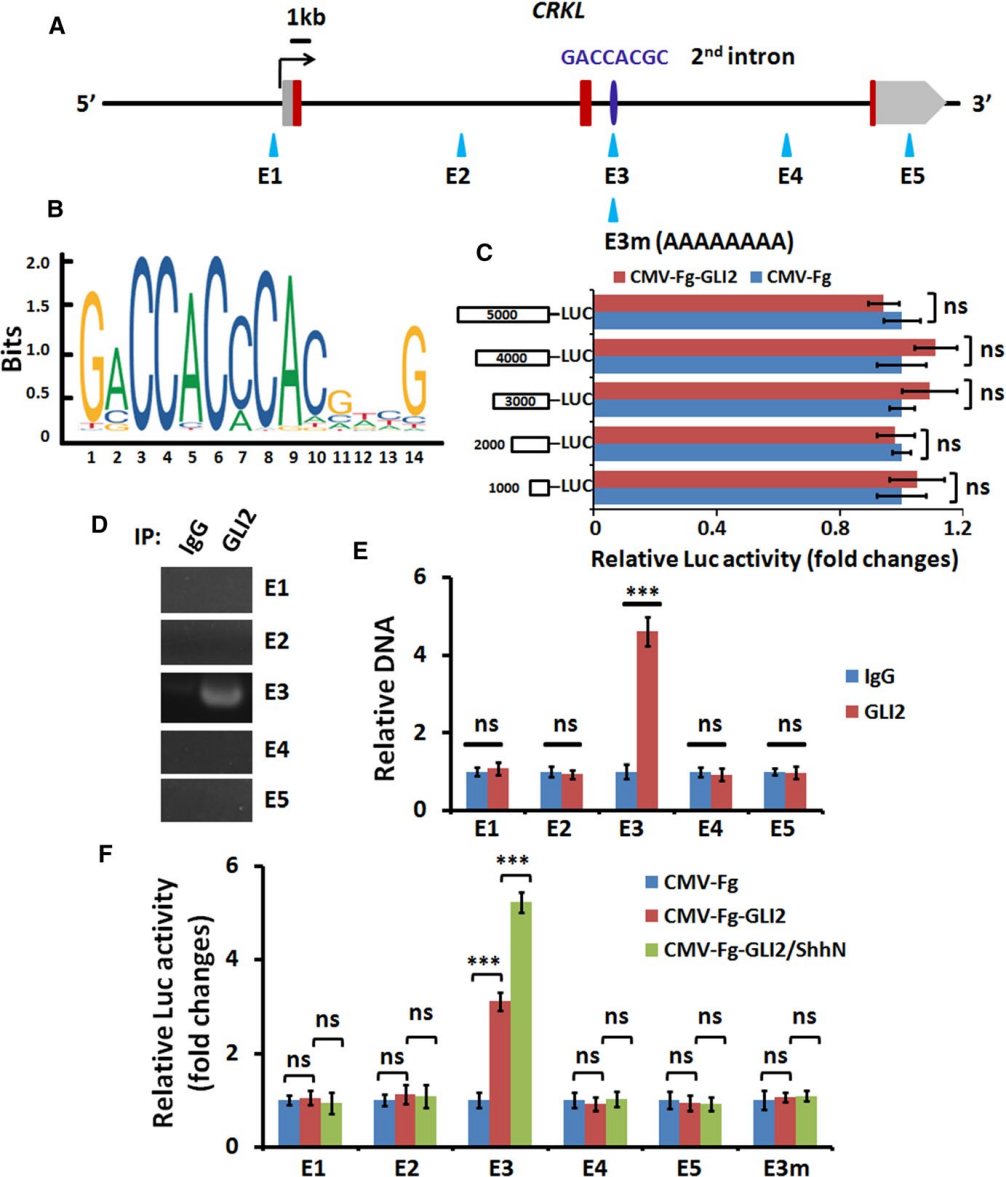
GLI2 binds directly to the 2nd intron of *CRKL* gene. A, Schematic view of *CRKL* locus and the presumptive GLI2 binding site (GCGACCAC) in the 2nd intron. E1 to E5 fragments contained about 200 base pairs. Luciferase reporters were connected to E1, E2, E3, E4 and E5 fragments. B, The conserved binding site of GLI2. C, Luciferase activity results from A549 cells transfected with indicated constructs. All luciferase reporters containing different promoter sequence were not responded to GLI2. Luciferase activities were normalized to Renilla luciferase activities. D, ChIP‐PCR results of A549 cell lysate immunoprecipitated by control IgG or GLI2 antibody. Of note, GLI2 antibody exclusively pulled down E3 fragment. E, ChIP‐qPCR data of A549 cell lysate immunoprecipitated by control IgG or GLI2 antibody. F, Luciferase activity results from A549 cells transfected with indicated constructs. Luciferase activities were normalized to Renilla luciferase activities. Notably, only E3‐luciferase could respond to GLI2. All values are mean ± SD (n = 3, ***P < .001 and ns, no significance)

To investigate whether GLI2 turns on *CRKL* expression directly, we examined the genome sequence of human *CRKL* and did not found any matched CLI2 binding site in the promoter region of *CRKL* (Figure 2A,B). However, a putative GLI2 binding site (GCGACCAC) was identified in the 2nd intron of *CRKL* (Figure 2A). We constructed five luciferase reporters which contain 1000 bp to 5000 bp sequence of *CRKL* promoter (Figure 2C). All the reporters were unable to respond to GLI2 (Figure 2C), suggesting that GLI2 activates *CRKL* expression not through its promoter. Thus, we focused our following study on the 2nd intron. To verify whether GLI2 binds the putative site in the 2nd intron, we carried out ChIP‐PCR and ChIP‐qPCR analyses and found that only GLI2 antibody pulled down E3 fragment, which contains the GLI2 binding site (Figure 2D,E). In addition, the luciferase reporter assay also revealed that only E3 fragment responded to GLI2 (Figure 2F). Importantly, mutating the consensus GLI2 binding site in E3 fragment abrogated its ability to respond to GLI2 (Figure 2F). Collectively, this evidence proved that GLI2 activates *CRKL* transcription through direct binding to its 2nd intron.

### 3.3 CRKL plays a critical role in GLI2‐driven cell proliferation and migration

**FIGURE 3:**
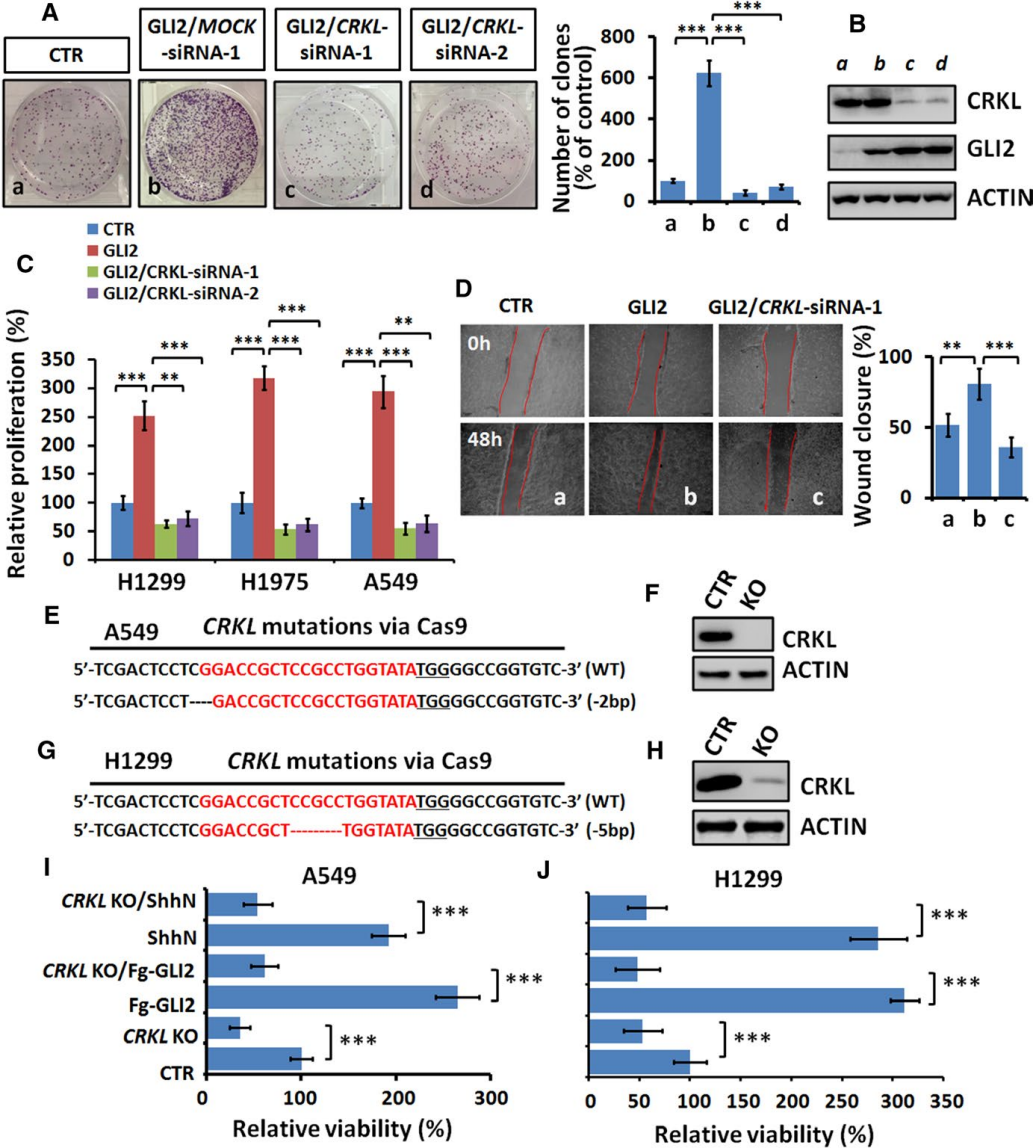
CRKL is critical for GLI2‐driven cell proliferation and migration. A, Clone numbers of A549 cells after indicated treatments. Quantification analyses were shown on right. B, Western blot analysis of A549 cells treated with indicated siRNAs. ACTIN acts as a loading control. C, MTT analyses of indicated LUAD cells under indicated transfections for 48 hours. Notably, GLI2 promoted NSCLC cells proliferation, which was counteracted by *CRKL* knockdown. D, Wound healing assays of A549 cells under indicated treatments. Quantification of wound closure at indicated time points was shown on right. E, Alignment of Sanger sequencing results of PCR amplicons from A549 cells. SgRNA targets were highlighted in red and the PAM sequence was underlined. F, Western blot analysis of CRKL expression of wild‐type (WT) and knockout (KO) A549 cells. G, Alignment of Sanger sequencing results of PCR amplicons from H1299 cells. H, Western blot analysis of CRKL expression of wild‐type (WT) and knockout (KO) H1299 cells. I, J, MTT results of WT or *CRKL* KO A549 and H1299 cells under indicated treatments. Notably, knockout of *CRKL* decreased cell proliferation and blunted GLI2‐driven cell proliferation. All values are mean ± SD (n = 3, **P < .01 and ***P < .001)

Our above data have clearly demonstrated that *CRKL* is a direct target gene of GLI2. *CRKL* is a well‐documented oncogene in several types of cancers, including NSCLC,^15^, ^16^ gastric cancer ^19^ and breast cancer.^20^ Thus, it is interesting to test whether GLI2 promotes NSCLC tumorigenesis through CRKL. The clone formation assay showed that overexpression of GLI2 indeed increased cell proliferation (Figure 3A), which was blunted by silencing *CRKL* (Figure 3A). The efficiencies of *CRKL* siRNAs were checked by Western blot assay (Figure 3B). On the other hand, MTT assays also revealed that GLI2‐induced cell viability could be counteracted by *CRKL* silence (Figure 3C). Furthermore, we showed that GLI2‐induced cell migration was neutralized by *CRKL* knockdown (Figure 3D). Overall, these results show that CRKL plays an indispensable role for GLI2‐induced cell proliferation and migration, at least in LUAD cells.

To confirm the role of CRKL in LUAD cell viability, we knocked out the endogenous *CRKL* using Cas9‐mediated gene editing in A549 cells. The Sanger sequencing identified a 2bp deletion cell line, which only expressed N‐terminal eight amino acid residuals of CRKL due to frameshift mutation (Figure 3E). The Western blot result further showed complete depletion of CRKL protein in this mutant cell line (Figure 3F). Consistently, knockout of *CRKL* apparently inhibited cell viability (Figure 3I). Moreover, the increased cell viability caused by GLI2 or ShhN was efficiently compromised by *CRKL* knockout (Figure 3I). We validated these results using another LUAD cell line, H1299 (Figure 3G,H and Figure 3J). These results showed that CRKL was essential for GLI2‐induced cell proliferation.

**FIGURE 4:**
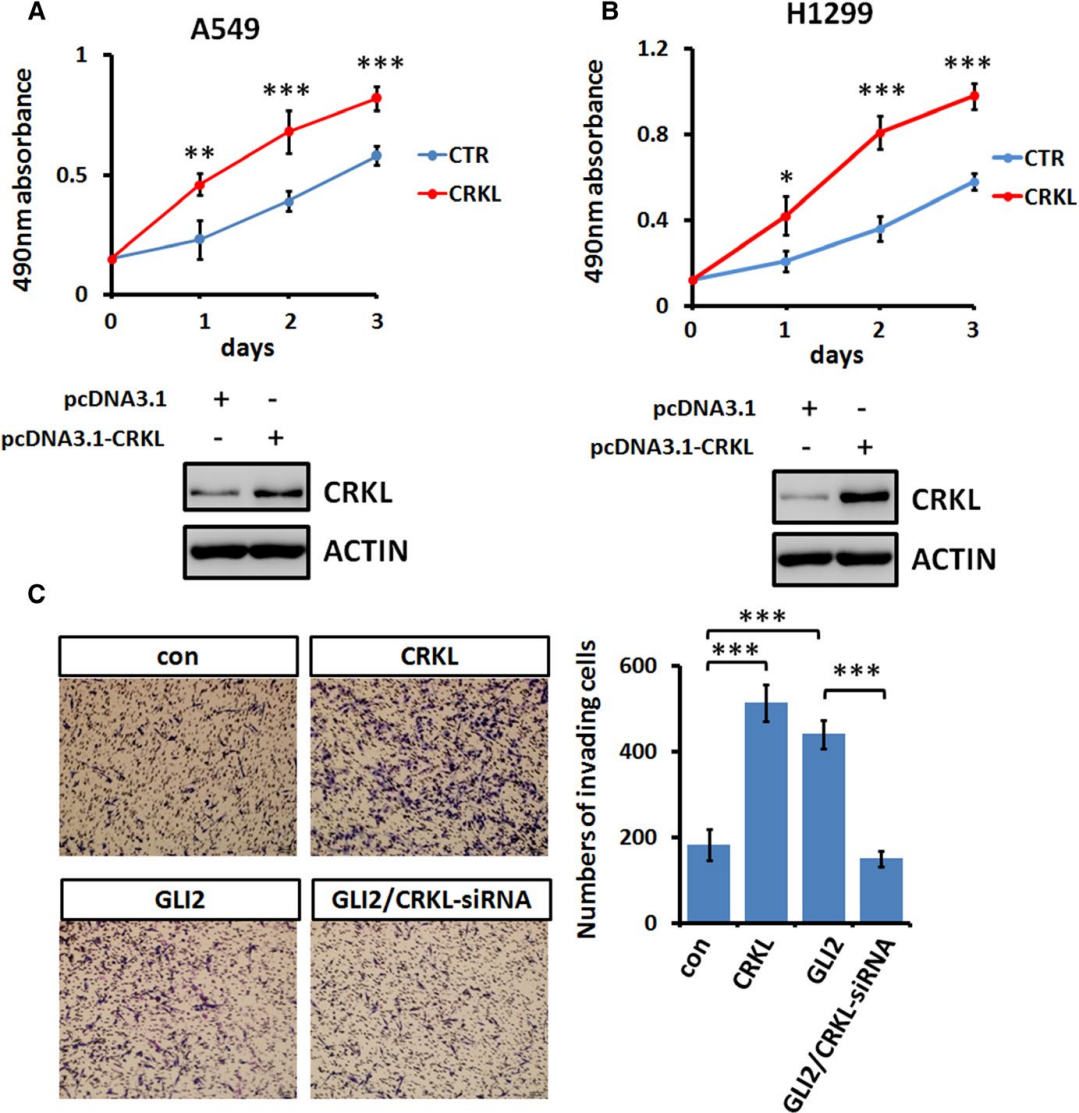
CRKL is sufficient to promote LAUD cell viability and invasion. A, Cell viability from A549 cells after indicated transfections. The expression of CRKL was examined by Western blot and showed below. B, MTT analyses of H1299 cells after indicated transfections. The expression of CRKL was examined by Western blot and showed below. C, Transwell results of A549 cells after indicated transfections. Quantification of invasive cell numbers was shown on right. All values are mean ± SD (n = 3, *P < .05, **P < .01 and ***P < .001)

On the other hand, we wanted to test whether *CRKL* overexpression is sufficient to promote LUAD cell viability and invasion. MTT results revealed that CRKL indeed enhanced cell viability of A549 and H1299 cells (Figure 4A,B). In addition, *CRKL* overexpression strongly promoted A549 cell invasion (Figure 4C). Consistently, GLI2 promoted A549 cell invasion, which was neutralized by CRKL knockdown (Figure 4C). Taken together, our findings have revealed that GLI2 promotes LUAD cell viability, migration and invasion through the transcriptional target *CRKL*.

### 3.4 CRKL knockout sensitizes LUAD cells to chemotherapies

**FIGURE 5:**
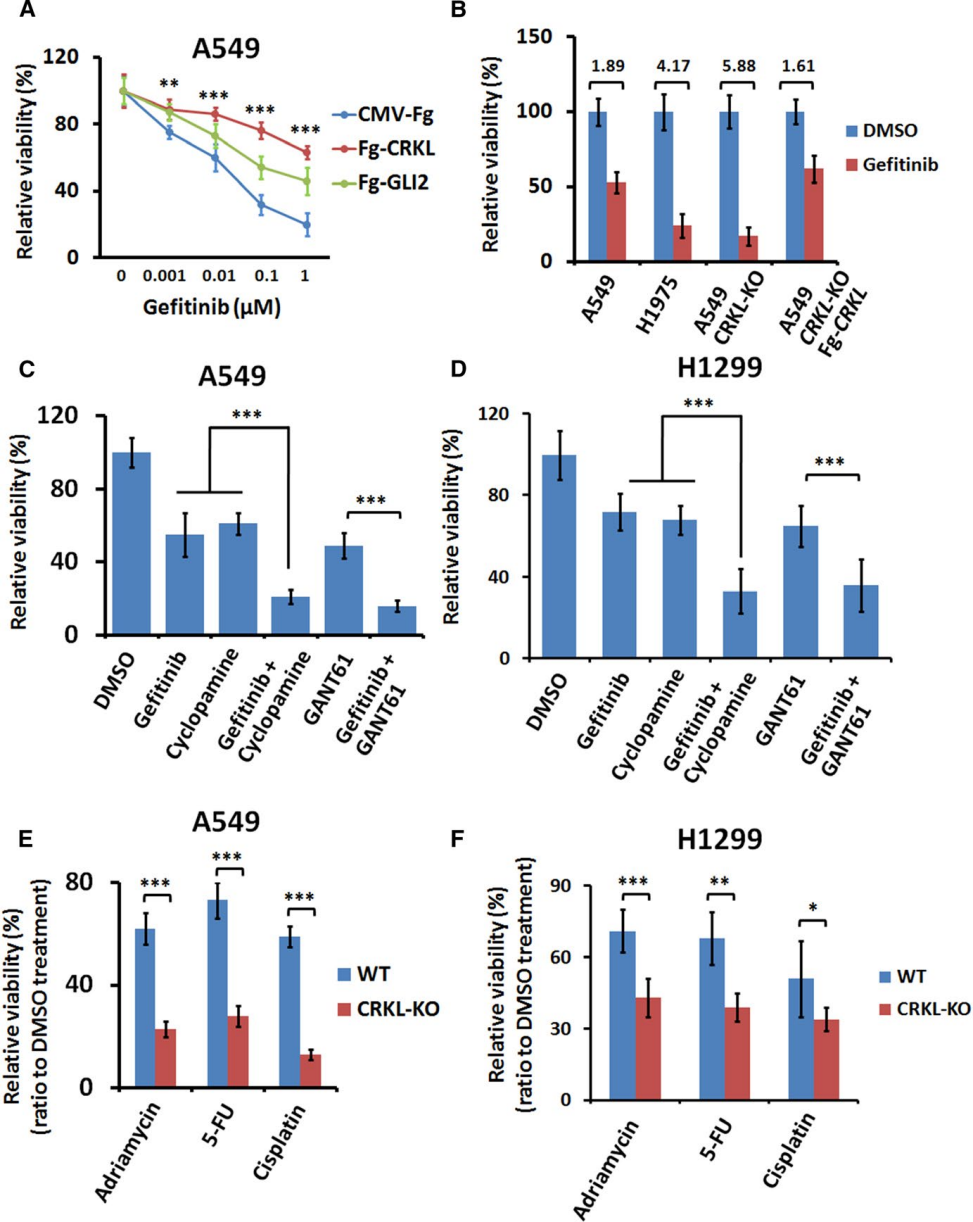
Knockout of *CRKL* sensitizes NSCLC cells towards EGFR‐TKI and chemotherapies. A, Cell proliferation from A549 cells treated with distinct concentrations of gefitinib. Both GLI2 and CRKL enhanced the resistance of A549 cells towards gefitinib. B, MTT analyses of indicated NSCLC cells treated with 0.01 μmol/L gefitinib or DMSO. Of note, *CRKL* KO A549 cells showed increased sensitivities to gefitinib compared with *CRKL* WT A549 cells. C, Cell viability results of A549 cells treated with indicated chemicals. D, Cell viability results of H1299 cells treated with indicated chemicals. E, F, Cell viability of *CRKL* WT or KO A549 and H1299 cells following treatment with adriamycin, 5‐FU or cisplatin for 24 hours. The data were normalized by control treatment with DMSO. All values are mean ± SD (n = 3, *P < .01, **P < .01 and ***P < .001)

In recent years, targeted therapy has emerged as a promising means to treat NSCLC.^21^ Epidermal growth factor receptor (EGFR) tyrosine kinase inhibitors (TKIs) have been used to treat NSCLC to inhibit EGFR pathway.^22^ However, most tumours initially responding to EGFR‐TKIs eventually recur as they acquire resistance.^23^ The mechanism of inducing EGFR‐TKIs resistance in NSCLC cells still remains unclear. The previous studies have demonstrated that *CRKL* overexpression promotes the viability of NSCLC cells in response to EGFR‐TKI gefitinib.^16^ In consistent with this, we found that CRKL indeed blunted the resistance of LUAD cells to gefitinib (Figure 5A). Intriguingly, GLI2 also compromised LUAD cell to gefitinib (Figure 5A). Compared with A549 cells, H1975 cells, which express less CRKL (Figure 1C), showed more sensitive to gefitinib (Figure 5B). Moreover, knockout of *CRKL* in A549 cells elevated the sensitivity to gefitinib (Figure 5B), which was overcome by introducing exogenous *CRKL* (Figure 5B). These results together suggest that Hh pathway improves NSCLC cells to EGRF‐TKIs, at least in part through CRKL. Consistently, combined treatment the NSCLC cells with gefitinib and Hh pathway inhibitor (cyclopamine or GANT61) significantly decreased the viability of A549 cells (Figure 5C) and H1299 cells (Figure 5D).

Chemotherapy is another means to treat NSCLC. We next wanted to test whether CRKL is involved in resistance to chemotherapies. To this end, we treated *CRKL* knockout (KO) and wild‐type (WT) A549 cells with various chemotherapeutical drugs including adriamycin, 5‐FU, cisplatin and DMSO as a control. After normalization by DMSO treatment, cell proliferation following different drugs treatment were concordantly lower in *CRKL* KO cells compared with WT cells (Figure 5E). Consistently, we got the similar results in H1299 cell (Figure 5F). Together, these data suggest that CRKL might contribute to the enhanced resistance to EGFR‐TKI and chemotherapies.

## 4 DISCUSSION

In this study, we identified that *CRKL* is a critical target of Hh‐GLI2 pathway to drive LUAD tumorigenesis. At first, overexpression of GLI2 promoted *CRKL* expression, whereas knockdown of GLI2 plays an opposite role. We also found that the expression of *GLI2* and *CRKL* was positively correlated in LUAD cell lines and patient samples. Mechanistically, GLI2 promoted *CRKL* expression via direct binding the 2nd intron of *CRKL* gene. Next, we figured out that CRKL was a necessary target for GLI2‐induced cell proliferation, migration and invasion. Furthermore, knockout of *CRKL* not only decreased cell proliferation, but also blunted GLI2‐driven tumorigenicity. At last, we showed that knockout of *CRKL* sensitized NSCLC cells to EGFR‐TKI and chemotherapies. Our finding identify CRKL is an important effector for GLI2‐driven NSCLC, and provide CRKL as a promising clinical target for NSCLC.

Albeit it is clear the oncogenic role of Hh pathway in NSCLC tumorigenesis, the inhibitor targeting Hh pathway for clinical therapeutics is still lack. Multifunction of Hh pathway limits its potential as a drug target. Thus, identification of the effector downstream of Hh pathway is urgent for Hh‐driven NSCLC. Here, we have demonstrated that the characteristic oncogene *CRKL* is the downstream effector of Hh‐GLI2 pathway, providing CRKL as a putative drug target for Hh‐related NSCLC. As a matter of fact, CRKL inhibitor PF‐114 has recently reported to induce tumour cell apoptosis.^24^ Given that our studies only uncover *CRKL* is a critical transcriptional target of Hh‐GLI2 pathway in NSCLC cells, it will be interesting to test whether this transcriptional regulation also exists in other types of cancer.

Resistance of cancer cells towards chemotherapies and EGFR‐TKIs is the major cause that leads to recurrence and poor survival of NSCLC patients. However, it is still unclear how do cancer cells acquire this resistance. In this study, we show that Hh pathway increases NSCLC cells resistance to gefitinib through the target CRKL. Treatment NSCLC cell with gefitinib and Hh pathway inhibitors significantly decreases cell proliferation, paving a new way to NSCLC therapy. In addition, knockout of *CRKL* apparently improves the sensitivity of NSCLC cells towards therapeutic drugs. Thus, combination of CRKL inhibitor and therapeutic drugs is possible an actionable means for NSCLC clinic treatment.

## CONFLICT OF INTEREST

The authors declare that they have no conflict of interest.

## AUTHOR CONTRIBUTIONS

**Xiaoming Liu:** Data curation (lead); Formal analysis (lead); Investigation (lead); Methodology (lead); Project administration (lead); Software (lead); Writing‐original draft (lead). **Ben‐tong Yu:** Formal analysis (supporting); Funding acquisition (supporting); Investigation (supporting); Methodology (supporting); Project administration (supporting). **Yan Hu:** Data curation (supporting); Formal analysis (supporting); Investigation (supporting); Methodology (supporting). **Kai Peng:** Funding acquisition (supporting); Investigation (supporting); Methodology (supporting); Project administration (supporting); Supervision (supporting); Validation (supporting). **Xin Gan:** Conceptualization (lead); Funding acquisition (lead); Project administration (lead); Supervision (lead); Validation (lead); Writing‐review & editing (lead).

## Data Availability

Some or all data and materials used during the study are available from the corresponding author by request.
